# Rates of referable eye disease in the Scottish National Diabetic Retinopathy Screening Programme

**DOI:** 10.1136/bjophthalmol-2013-303948

**Published:** 2014-03-05

**Authors:** H C Looker, S O Nyangoma, D T Cromie, J A Olson, G P Leese, M W Black, J Doig, N Lee, R S Lindsay, J A McKnight, A D Morris, D W M Pearson, S Philip, S H Wild, H M Colhoun, on behalf of the Scottish Diabetes Research Network Epidemiology Group and the Scottish Diabetic Retinopathy Collaborative

**Affiliations:** 1 University of Dundee, Dundee, UK; 2 NHS Lanarkshire, Glasgow, UK; 3 NHS Grampian, Aberdeen, UK; 4 Ninewells Hospital & Medical School, Dundee, UK; 5 Diabetic Retinopathy Screening Collaborative, NHS Highland, UK; 6 Forth Valley Royal Hospital, Edinburgh, UK; 7 University of Glasgow, Glasgow, UK; 8 Western General Hospital, Edinburgh, UK; 9 University of Edinburgh, Edinburgh, UK; 10 Aberdeen Royal Infirmary, Aberdeen, UK; 11 Grampian Diabetes Research Unit, NHS Grampian, Aberdeen, UK

**Keywords:** Epidemiology, Retina, Macula

## Abstract

**Aims:**

Diabetic retinopathy screening aims to detect people at risk of visual loss due to proliferative diabetic retinopathy, but also refers cases of suspected macular oedema (maculopathy). At the introduction of screening, ophthalmology was concerned that referral rates would be unmanageable. We report yield of referable disease by referral reason for the first 5 years of the programme.

**Methods:**

We extracted screening results from a nationwide clinical diabetes database to calculate annual referral rates to ophthalmic clinics. We used logistic regression to examine associations between clinical measures and referable disease.

**Results:**

182 397 people underwent ≥1successful retinal screening between 2006 and 2010. The yield of referable eye disease was highest in the first 2 years of screening (7.0% and 6.0%) before stabilising at ∼4.3%. The majority of referrals are due to maculopathy with 73% of referrals in 2010 based on a finding of maculopathy.

**Conclusions:**

The commonest cause for referral is for suspected macular oedema (maculopathy). Referral rates for retinopathy have stabilised, as predicted, at relatively low rates. However, ophthalmology workload continues to rise as new treatment options (ie, monthly intraocular injections) have unexpectedly increased the impact on ophthalmology. A review of the screening referral path for maculopathy may be timely.

## Introduction

Diabetic retinopathy (DR) is one of the leading causes of visual impairment in the UK,^1^ and treatment to prevent vision loss requires early detection and careful monitoring to be most effective.^2^ Following a Health and Technology Board Assessment, Scotland launched a national Scottish DR Screening service (DRS) in 2006. The principal aim of screening was to prevent vision loss due to proliferative DR (PDR), with individuals identified as high risk being referred to eye clinics for assessment and further management. Maculopathy (MAC) did not meet the requirements to justify a screening programme, but as it can be detected on retinal photography, a MAC grading is included in the DRS protocols with referral based on MAC as well as DR grades. Based on pilot data from the Grampian region the expected long-term referral rate from the programme was expected to be 3–4%. This workload was thought to be manageable within existing ophthalmology provision.^3^ The aim of this study is to report the yield of referable disease over the first 5 years of the DRS (2006–2010), a measure important for health service planning, and to estimate the burden due to referable MAC.

## Methods

### DRS protocols

The national roll out of the DRS began in 2006 to improve availability of high quality retinal screening in Scotland^4^ attaining nationwide coverage in 2007. Patients eligible for screening are identified via the national diabetes registry—the Scottish Care Information-Diabetes Collaboration (SCI-DC) database—which automatically captures data on people with a diagnosis code for diabetes and has an estimated current coverage of >99% of the Scottish population with diagnosed diabetes mellitus (DM). All new registrants on SCI-DC who are aged ≥12 years are automatically registered as new patients on the DRS database and this triggers their first appointment. Those who are already attending eye clinics for DM-related eye disease, and those who are too unfit or frail for screening, are suspended from the screening programme with suspensions reviewed at least every 3 years. The screening examination involves a single central 45° field digital photograph with mydriasis if required, and with centralised grading.^3^ When photographic images are ungradable, slit-lamp examination is undertaken within the screening programme. Slit-lamp examination gradings were not available for all health boards for the whole time course of the study, but where slit-lamp data are available it is included in the results (within this study 4.2% of grades were from slit-lamp exams). The use of a single central field for detection of sight-threatening retinopathy has been validated.^5–8^ The programme includes internal and external quality control protocols to ensure the quality of the photographs and the grading.^9^

### DRS grading

The grading system in Scotland provides a DR grade and a MAC grade (see online supplementary table S1). Action is determined by the most severe finding in the worst eye with grades of R3 (referable background retinopathy), R4 (proliferative retinopathy) or M2 (referable MAC) triggering referral to a specialist eye clinic. Previous analyses from the pilot phase of the DRS reported an overall referral rate for eye disease of ∼3%.^3^ ^10^ DRS grading data is automatically uploaded into the SCI-DC database. We use the results for the worst eye to determine grading in this study.

### Data sources

We used an anonymised extract of SCI-DC data that includes data on all individuals alive and registered on SCI-DC as of 31 May 2008 (the most recent SCI-DC extract data available) which has updated DRS data up to 31 December 2010. Data was extracted from the SCI-DC audit server by an automated process and then cleaned and anonymised prior to use in this study.

### Study population

All individuals age >12 years who were registered on SCI-DC with a diagnosis of diabetes before 31 May 2008 and had at least one successful DRS examination were included in this study.

### Covariate data

Diabetes type was based on the type listed in SCI-DC by clinicians. Additionally, we employed an algorithm to identify individuals at high risk of being mislabelled, based on age of diagnosis and prescribed treatment. The date of diagnosis for DM was based on the diagnosis date entered into SCI-DC by clinicians, taking the earliest date where multiple dates were entered. This diagnosis date was checked against multiple data sources including prescription and hospital discharge records for any evidence of diabetes prior to the SCI-DC date of diagnosis. Where there is evidence of DM prior to the recorded date of diagnosis we used the earliest date available.

To determine the risk factor profile for cases of referable eye disease we considered risk factor data for those examined in 2008. Measures of Body Mass Index (BMI), HbA1c, serum total cholesterol, systolic and diastolic blood pressure and smoking status were selected from SCI-DC taking the measure made nearest to the screening exam. In order to limit missing risk factor data while retaining a temporal relationship between measure and screening we used a series of cut-points (HbA1c ±180 days, blood pressure ±90 days and BMI or smoking status ±1085 days). If no measure was available the data was considered to be missing. We used prescription data to determine diabetes treatment at the time of the screening examination.

### Statistical methods

The proportion of screened individuals with referable eye disease was calculated by year. Where an individual had more than one screening examination in a year the worst result was used. We used univariate and multivariate logistic regression models to examine the role of DM type, age, sex and diabetes duration along with other covariates on risk for referable eye disease. All statistical analysis was undertaken using SAS v.9.2 (Cary, North Carolina, USA). We used a p value of <0.05 to indicate statistical significance.

## Results

**Figure 1:**
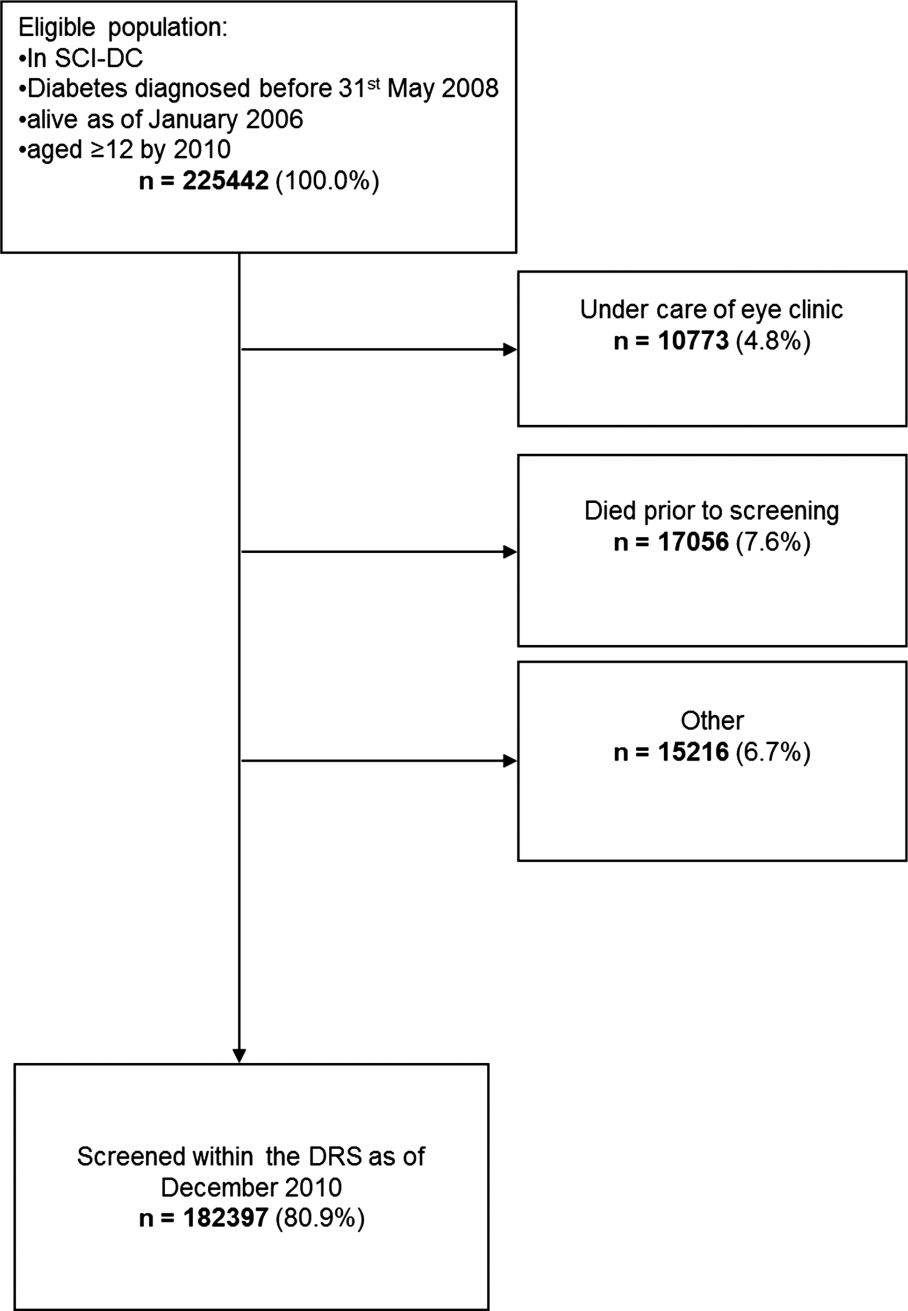
Flow diagram to demonstrate use of DRS among individuals from SCI-DC during the period 1 January 2006–31 December 2010. DRS, Diabetic Retinopathy Screening service; SCI-DC, Scottish Care Information-Diabetes Collaboration.

There were 225 442 individuals eligible for inclusion in this study. During the course of the study 187 822 (83.3%) were screened at least once by the DRS, with 182 397 having at least one gradable result (figure 1). Demographics for the study population are shown in table 1.

**Table 1:** Baseline demographics for population

	Frequency/median
Male sex	100 733 (55.2%)
Diabetes type (%)	
Type 1 diabetes	20 521 (11.3)
Type 2 diabetes	161 135 (88.3)
Other/unknown type	741 (0.4)
Year first screened (%)	
2006	39 463 (21.6)
2007	89 720 (49.2)
2008	33 777 (18.5)
2009	11 142 (6.1)
2010	8295 (4.6)
Age at first screen (years)	64 (54–73)
Diabetes duration at first screen (years)	5.7 (2.4–10.8)

### Yield of referable eye disease

We included data on 560 362 screening episodes resulting in gradable images between 2006 and 2010. Slit-lamp examinations accounted for 23 311 (4.8%) of the data. Each individual had a median of three (IQR 2–4) successful screens during the study period and this did not differ by DM type (table 2). The yield of referable eye disease was greatest in the first 2 years of the programme where 7.0% and 6.0% of individuals successfully screened in the DRS had referable disease in 2006 and 2007, respectively. Over time, the proportion of people attending who had attended at least one previous screen by the DRS increased from 23.5% of screens in 2007 to >90% of screens in 2009 and 2010. Referral rates were highest for first-time attendees with 6.9% of first screens finding referable eye disease compared to 3% for people attending their 4th or 5th screen.

**Table 2:** Yield of referable eye disease by year of screening

	Yield of referable eye disease n (%)				
	2006 (n=39 463)	2007 (n=117 348)	2008 (n=128 897)	2009 (n=128 203)	2010 (n=130 170)
No eye disease	24 734 (62.7)	76 818 (65.5)	87 184 (67.6)	85 676 (66.8)	88 019 (67.6)
Non-referable eye disease	11 950 (30.3)	33 524 (28.6)	36 197 (28.1)	36 956 (28.8)	36 500 (28.0)
Referable eye disease	2779 (7.0)	7006 (6.0)	5516 (4.3)	5571 (4.3)	5651 (4.3)
Referable maculopathy alone	1666 (4.2)	4587 (3.9)	3805 (3.0)	3993 (3.1)	4107 (3.2)
Referable background retinopathy±maculopathy	716 (1.8)	1452 (1.2)	978 (0.8)	800 (0.6)	751 (0.6)
Proliferative retinopathy±maculopathy	397 (1.0)	967 (0.8)	733 (0.6)	778 (0.6)	793 (0.6)

Referable eye disease comprises referable maculopathy (M2 on the DRS grading scheme), preproliferative retinopathy (R3 on the DRS grading scheme) and proliferative retinopathy (R4 on the DRS grading scheme).

DRS, Diabetic Retinopathy Screening service.

For 2008 and onwards, the yield was stable at 4.3%. For all years, the yield for referable MAC was higher than that for referable DR accounting for between 60% of referrals in 2006 to 73% in 2010. Overall, the referral rates were higher for those screened by slit-lamp (7.2%) than those screened by retinal photography (5.1%).

### Referable eye disease by type of diabetes

We excluded 741 individuals from this analysis as they had neither type 1 (T1DM) nor type 2 (T2DM) (eg, MODY, pancreatic causes, etc; table 3). There were 20 521 people with T1DM and 161 135 people with T2DM (table 2). The prevalence of referable eye disease was consistently higher among those with T1DM compared to those with T2DM (table 3). The proportion of referrals due to MAC alone were similar for both types of DM in 2006 (59.0% for T1DM and 60.3% for T2DM), but in subsequent years the proportion rose among the T2DM population to 76.1% in 2010 compared to 63.2% of the referrals among the T1DM population.

**Table 3:** Yield of referable eye disease for individuals with type 1 or type 2 diabetes by year of screening

	Type 1 diabetes					Type 2 diabetes				
	2006	2007	2008	2009	2010	2006	2007	2008	2009	2010
No eye disease	1886 (38.0)	5368 (41.3)	5872 (43.8)	5675 (41.7)	5765 (41.6)	22 750 (66.2)	71 156 (68.4)	80 961 (70.4)	79 656 (69.8)	81 894 (70.7)
Non-referable eye disease	2346 (47.2)	5943 (45.8)	6146 (45.9)	6458 (47.5)	6577 (47.4)	9563 (27.8)	27 491 (26.4)	29 951 (26.0)	30 393 (26.6)	29 812 (25.7)
Referable eye disease	735 (14.8)	1676 (12.9)	1377 (10.3)	1465 (10.8)	1521 (11.0)	2038 (5.9)	5315 (5.1)	4128 (3.6)	4087 (3.6)	4113 (3.6)
Referable maculopathy alone	434 (8.7)	1023 (7.9)	856 (6.4)	945 (7.0)	962 (6.9)	1229 (3.6)	3555 (3.4)	2941 (2.6)	3034 (2.7)	3131 (2.7)
Referable background retinopathy±maculopathy	161 (3.2)	318 (2.4)	237 (1.8)	202 (1.5)	212 (1.5)	554 (1.6)	1129 (1.1)	740 (0.6)	596 (0.5)	537 (0.5)
Proliferative retinopathy±maculopathy	140 (2.8)	335 (2.6)	284 (2.1)	318 (2.3)	347 (2.5)	255 (0.7)	631 (0.6)	447 (0.4)	457 (0.4)	445 (0.4)
Total (100%)	4967	12 987	13 395	13 598	13 863	34 351	103 962	115 040	114 136	115 819

Referable eye disease comprises referable maculopathy (M2 on the DRS grading scheme), preproliferative retinopathy (R3 on the DRS grading scheme) and proliferative retinopathy (R4 on the DRS grading scheme).

DRS, Diabetic Retinopathy Screening service.

### Risk factor profile for referable eye disease

Table 4 shows the risk factor profile by retinal screening grade. Regardless of diabetes type, compared to the no retinopathy group, those with referable disease were more often male, with longer DM duration, higher HbA1c, and higher systolic blood pressure. Additionally, for people with T1DM, referable disease was also associated with older age, higher BMI, higher diastolic blood pressure and higher prevalence of smoking. By contrast, in T2DM, referable disease was also associated with lower prevalence of smoking, lower BMI and greater use of insulin therapy.

**Table 4:** Risk factors for referable eye disease for individuals with type 1 or type 2 diabetes in 2008

	No retinopathy or maculopathy	Non-referable retinopathy	Referable maculopathy only	Referable retinopathy±referable Maculopathy	p Value No retinopathy vs Referable maculopathy	p Value No retinopathy vs referable retinopathy	p Value Referable maculopathy vs referable retinopathy
Type 1 diabetes	5872	6146	856	521			
Male sex*	3122 (53.2%)	3415 (55.6%)	498 (58.2%)	331 (63.5%)	0.006	<0.001	0.049
Age (years)	35.1±16.9	42.6±14.5	43.8±13.8	41.8±12.3	<0.001	<0.001	0.046
Diabetes duration (years)†	7.7 (3.6–14.4)	19.6 (12.3–28.9)	20.7 (15.4–28.2)	23.1 (17.0–30.0)	<0.001	<0.001	<0.001
HbA1c (%)	8.5±1.7	8.7±1.6	9.1±1.7	9.4±1.9	<0.001	<0.001	0.002
Systolic blood pressure (mm Hg)	126.5± 16.4	130.2±17.1	132.5±15.8	132.9±18.1	<0.001	<0.001	0.409
Diastolic blood pressure (mm Hg)	73.8±10.0	74.3±9.9	76.3±10.2	76.0±10.8	<0.001	<0.001	0.351
BMI (kg/m^2^)	25.9±5.4	27.2±5.0	27.8±5.1	26.8±5.3	<0.001	0.003	0.009
Current smoking*	851 (15.1%)	1200 (20.7%)	159 (20.0%)	114 (23.8%)	0.048	0.003	0.130
Type 2 diabetes	80 961	29 951	2941	1187			
Male sex*	44 615 (55.1%)	17 535 (58.6%)	1672 (56.9%)	708 (59.7%)	0.062	0.002	0.100
Age (years)	64.8±11.5	65.3±11.4	64.8±11.5	65.1±11.5	0.710	0.220	0.293
Diabetes duration (years)†	4.8 (2.4–8.1)	7.8 (4.1–12.8)	11.3 (6.2–16.2)	13.2 (7.4–18.1)	<0.001	<0.001	<0.001
HbA1c (%)	7.3±1.4	7.6±1.6	8.1±1.8	8.4± 1.8	<0.001	<0.001	<0.001
Systolic blood pressure (mm Hg)	134.3±15.9	136.3±17.1	138.4±18.0	139.9±20.6	<0.001	<0.001	0.043
Diastolic blood pressure (mm Hg)	75.7±10.2	75.3±10.2	75.8±10.4	75.7±10.4	0.886	0.818	0.837
BMI (kg/m^2^)	31.6±6.3	31.4±6.3	31.2±6.3	31.9±6.5	0.001	0.037	<0.001
Current smoking*	10 306 (14.5%)	3797 (14.6%)	310 (12.1%)	129 (12.1%)	<0.001	0.015	0.874
Diabetes treatment*					<0.001	<0.001	<0.001
No medication	25 464 (31.5%)	5729 (19.1%)	321 (10.9%)	111 (9.4%)			
Oral agents only	49 332 (60.9%)	18 593 (62.1%)	1652 (56.2%)	579 (48.8%)			
Insulin	6165 (7.6%)	5629 (18.8%)	968 (32.9%)	497 (41.9%)			

Data are means with SDs except for * which are frequencies, and † which are medians with IQR.

Referable eye disease comprises referable maculopathy (M2 on the DRS grading scheme), preproliferative retinopathy (R3 on the DRS grading scheme) and proliferative retinopathy (R4 on the DRS grading scheme).

p Values are for multivariate logistic regression models including all covariates and screening modality (retinal photography or slit-lamp).

Covariate data was complete for 78 814 (61.4%) of the individuals. Blood pressure data was missing for 31 767 (24.7%) Smoking data was missing for 15 101 (11.8%) HbA1c data was missing for 9946 people (7.7%) and BMI data was missing for 7025 people (5.5%).

BMI, Body Mass Index; DRS, Diabetic Retinopathy Screening service.

Compared to people referred for DR, people referred with MAC had shorter DM duration and lower HbA1c values. Additionally, among people with T1DM, MAC referrals tended to be for older women with higher BMI than referrals for DR, whereas for people with T2DM, MAC referral was associated with lower BMI and lower systolic blood pressure,

In multivariate models restricted to those with no missing data (n=78 814) including all covariates and screening mode male sex, longer DM duration and higher HbA1c remained significantly associated with referable disease regardless of DM type. For T2DM, higher blood pressure, lower prevalence of smoking and higher prevalence of insulin use were also associated with referable disease.

In a univariate logistic regression model for the presence of any referable eye disease using data for 2008, the OR for T1DM compared to T2DM was 3.08 (95% CI 2.89 to 3.28, p<0.001), with risk seemingly mediated principally via DM duration (OR for referral due to T1DM 0.81 (95% CI 0.73 to 0.91) after adjustment for age, sex, DM duration and screening method). T1DM was a positive risk factor for referral due to PDR even after adjustment for age, sex and DM duration and screening method (OR=1.35, OR 1.06 to 1.73, p=0.017). However, due to the limited overlap between age and DM duration ranges for the DM types (table 4) these models may not fully account for the covariates.

## Discussion

The yield of referable eye disease within the DRS was at its highest during the first 2 years of the programme (2006–2007) when 7% of successfully screened individuals were referred to the eye clinic. Since 2008, the annual referral rate has been stable at 4.3% (∼5500 referrals per year). This suggests that the DRS successfully screened people who had been missed by earlier non-national screening programmes and has now reached a steady level. This workload should be manageable within existing ophthalmology provision.^3^ In keeping with this, we showed that referable disease was more commonly found at an individual's initial screening which reflects our earlier work on screening intervals.^11^ However, despite the screening programme being designed for the prevention of sight loss due to PDR, 73% of referrals in 2010 were for suspected macular oedema. That so many of the referrals are due to suspected macular oedema is important as we are facing a shift in treatment options for macula oedema. While patients with diabetic macular oedema used to be routinely offered a finite course of laser treatment, patients are now being offered monthly, indefinite, intraocular injections. As laser for macular oedema cannot restore vision, it was important to offer treatment to patients, even when asymptomatic. However, new treatments restore vision in many, albeit at the cost of indefinite monthly intraocular injections.^12^ ^13^ This new development questions the need to detect patients with macular oedema while they are still asymptomatic, suggesting the way the DRS deals with macula oedema deserves review. Of note, this study is based on the finding of referable disease and, thus, is not influenced by clinical factors that may influence whether an individual attends eye clinic subsequent to screening.

Strengths of the study are the inclusion of data from a national DR screening programme. The DRS methodology has also been validated for detection of sight-threatening DR,^5–8^ and while there have been adjustments to the grading algorithm over time these changes have not effected the referable disease gradings which have remained consistent. As Scotland also has a means for linking an individual's medical data from a variety of sources (via a unique health identifier the CHI number) we can incorporate clinical data to the DR screening data. There are also weaknesses to the study. Primarily, the current data extract only includes individuals diagnosed with DM prior to 31 May 2008. Therefore, for the years 2008–2010, we are missing data on people with DM diagnosed after 31 May 2008. We do not believe this will have greatly altered the prevalence of referable eye disease reported in the study, as referable disease is very rare in newly diagnosed people with T1DM,^14^ and in this population <2% of individuals with newly diagnosed T2DM have referable retinopathy at their first screening examination.^15^ Thus, it is probable that our estimate of yield is higher than the true. We also cannot report outcomes of referral as we lack data from eye clinics.

The reported overall referral rates are in line with those proposed during the pilot stage of the DRS 3 and in keeping with reports of regional data from England.^16^ The principle risk factors we identified as associated with referable eye disease at screening are in keeping with those reported for DR as a whole, and are independent of the screening method (slit-lamp vs photography), that is, hyperglycaemia, hypertension, DM duration^17^ and insulin therapy for T2DM.^18^ The role of BMI as a risk factor for retinopathy is less clear-cut with positive associations reported in T1DM^19^ and T2DM,^20^ ^21^ while the association we report between lower BMI and referable MAC in T2DM is consistent with findings from the WESDR.^18^ Similarly, there is inconsistent data for the role of smoking and referable eye disease.^22^ ^23^

In the USA, the prevalence of sight-threatening retinopathy among people with T1DM is as high as 30%.^24^ In the past, for people with DM of >20 years duration, the prevalence of any DR among people with T1DM approaches 100% 14 compared to ∼60% in people with T2DM.^18^ ^24^ However, recent data from Wisconsin shows a lower prevalence of DR at 20 years DM duration for people diagnosed with T1DM in 1987–1992 compared with those diagnosed prior to 1980.^25^

While we may see reductions in the prevalence of referable disease over time due to improvements in DM management, the current referral rates indicate a steady rate of new referrals from the national screening programme with the majority a result of referable MAC rather than of referable DR. A review of the referral pathway for MAC to better reflect the changing treatment options could have a significant impact on the screening pathway and on ophthalmology workload.

## Supplementary Material

Web supplement
