# Supplementary material for: Rates of referable eye disease in the Scottish National Diabetic Retinopathy Screening Programme
Source: Br J Ophthalmol. 2014 Mar 5;98(6):790–5. doi: 10.1136/bjophthalmol-2013-303948 (PMC4033179; doi:10.1136/bjophthalmol-2013-303948)
Supplement: Web supplement [file bjophthalmol-2013-303948-s1.pdf]

Supplementary Table 1 Retinopathy and maculopathy grading scheme for the Scottish Diabetes Retinopathy Screening Service [4]

Code	Description
Retinopathy (DR)	
R0	No visible diabetic retinopathy anywhere
R1(Mild)	Background diabetic retinopathy (BDR) – mild<br>• At least one dot haemorrhage or microaneurysm with or without hard exudates
R2(Observable)	BDR - moderate<br>• Four or more blot haemorrhages (i.e. ≥ Airlie House standard photograph 2a) in one hemi-field only(Inferior and superior hemi-fields delineated by a line passing through the centre of the fovea and optic disc)
R3(Referable)	BDR - severe<br>Any of the following features:<br>• Four or more blot haemorrhages (i.e. ≥ Airlie House standard photograph 2a) in both inferior and superior hemi-fields<br>• Venous beading(≥Airlie House standard photograph 6a)<br>• Intraretinal microvascular abnormalities (IRMA)(>=Airlie House standard photograph 8a)
R4(Proliferative)	Proliferative diabetic retinopathy (PDR)<br>Any of the following features<br>• New vessels<br>• Vitreous haemorrhage
Maculopathy (MAC)	
M1(Observable)	Lesions within a radius of >1 but <2 disc diameters of the centre of the fovea<br>• Any hard exudates
M2(Referable)	Lesions within a radius of <1 disc diameter of the centre of the fovea<br>• Any blot haemorrhages<br>• Any hard exudates<br>• Macula Oedema
